# Transcriptomic Analysis Reveals Fibroblast Growth Factor 11 (FGF11) Role in Brown Adipocytes in Thermogenic Regulation of Goats

**DOI:** 10.3390/ijms241310838

**Published:** 2023-06-29

**Authors:** Tingting Jiang, Duo Su, Xin Liu, Yan Wang, Linjie Wang

**Affiliations:** 1 Key Laboratory of Livestock and Poultry Multi-Omics, Ministry of Agriculture and Rural Affairs, College of Animal and Technology, Sichuan Agricultural University, Chengdu 611130, China; jiangtingting2021@163.com (T.J.); suduo0821@163.com (D.S.); 2020202011@stu.sicau.edu.cn (X.L.); wangyan8108@sicau.edu.cn (Y.W.); 2 Farm Animal Genetic Resources Exploration and Innovation Key Laboratory of Sichuan Province, Sichuan Agricultural University, Chengdu 611130, China

**Keywords:** brown adipose tissue, FGF11, thermogenesis, goats, UCP1

## Abstract

Brown adipose tissue (BAT) is the main site of adaptive thermogenesis, generates heat to maintain body temperature upon cold exposure, and protects against obesity by promoting energy expenditure. RNA-seq analysis revealed that FGF11 is enriched in BAT. However, the functions and regulatory mechanisms of FGF11 in BAT thermogenesis are still limited. In this study, we found that FGF11 was significantly enriched in goat BAT compared with white adipose tissue (WAT). Gain- and loss-of-function experiments revealed that FGF11 promoted differentiation and thermogenesis in brown adipocytes. However, FGF11 had no effect on white adipocyte differentiation. Furthermore, FGF11 promoted the expression of the UCP1 protein and an EBF2 element was responsible for UCP1 promoter activity. Additionally, FGF11 induced UCP1 gene expression through promoting EBF2 binding to the UCP1 promoter. These results revealed that FGF11 promotes differentiation and thermogenesis in brown adipocytes but not in white adipocytes of goats. These findings provide evidence for FGF11 and transcription factor regulatory functions in controlling brown adipose thermogenesis of goats.

## 1. Introduction

Adipose tissues are broadly classified as white adipose tissue (WAT), brown adipose tissue (BAT), and beige adipose tissue [1]. WAT is specialized in lipid storage and mobilization. White adipocytes provide metabolic fuel through lipolysis and release fatty acids into the blood [2]. In contrast to WAT, BAT not only dissipates energy as heat via non-shivering thermogenesis in response to cold exposure [3], but also serves as a metabolic sink for glucose, fatty acids, and branched-chain amino acids to improve metabolic health [4,5,6]. BAT can also secrete autocrine and paracrine factors, many of which can regulate BAT recruitment and thermogenic activity [7]. This thermogenic process consumes large amounts of glucose and fatty acids. Similarly, beige adipocytes can be recruited in WAT via β-adrenergic signaling and consume glucose and fatty acids when exposed to cold [8,9,10]. It has been suggested that the thermogenic capacity of brown and beige adipose tissue is due to their high mitochondrial numbers and high levels of uncoupling protein 1 (UCP1) expression [11,12].

Recent studies have confirmed the presence of BAT in the supraclavicular region [13], deep neck, and the perirenal region in adult humans [14,15]. The interscapular BAT of mice maintains the characteristics of brown adipose and possesses thermogenic capacity in adulthood. A previous study has demonstrated that UCP1 is prominently enriched in the sternal adipose tissue of sheep, suggesting its potential contribution to the thermogenic response associated with feeding [16]. However, the roles of BAT function in adults are still unclear. The major BAT depots of lambs and human infants are located in the supraclavicular/neck, pericardial, and perirenal regions, which convert from BAT to WAT in adulthood [17]. However, the interscapular BAT of mice maintains the characteristics of brown adipose and possesses thermogenic capacity in adulthood [18]. Our previous study showed that BAT in goats is recruited at birth and converted to WAT at a month after birth [19,20]. Newborn lambs are usually born in a cold environment, and lambs have low-fat coverage and a high surface area to body weight ratio, which aggravates the loss of heat [21]. Thus, thermogenesis in lamb adipose is necessary to maintain body temperature. Our recent study found that BAT thermogenesis is induced in newborn goats during cold exposure [22]. The thermogenesis capacity of BAT in newborn goats was improved after L-carnitine treatment, which promotes BAT differentiation and thermogenesis through the AMPK pathway [23,24].

The FGF family includes a total of 23 members (FGF1–FGF23), which are divided into intracellular FGFs (iFGFs), canonical FGFs, paracrine FGFs, and hormone FGFs [25]. Among the FGF family, several members have been proved to play an important role on adipose tissue thermogenesis and differentiation. It has been proven in rodents that the expression of FGF6 and FGF9 is induced in BAT by exercise and cold, and FGF6 and FGF9 regulate UCP1 independent of brown adipogenesis through the FGFR3–FGF6/9-PGE2–UCP1 pathway [26]. The expression of FGF21 is markedly increased in the adipose tissue upon cold stimulation and regulates UCP1 and PGC1α expression, thereby modulating body defense against cold stimuli [27]. FGF16 promotes mitogenic activity by binding FGF receptor 4 in BAT [28]. Sympathetic output to brown adipose and causes obesity were impaired in FGF13 knockout mice [29]. FGF4 enhances fatty oxidation through the FGFR4–AMPK–Caspase 6 signaling pathway with reduced hepatocyte apoptosis, thereby protecting the liver from nonalcoholic fatty liver damage [30]. FGF1 is a key regulator of human adipogenesis. FGF1 can promote human preadipocyte proliferation and differentiation, but not in 3T3-L1 cells [31].

Analysis of our transcriptome data showed that within the FGF family, only FGF9 and FGF11 exhibited upregulated expression in BAT. A previous study has demonstrated that FGF9 is confirmed as a positive regulator to promote the expression of thermogenic genes in brown adipose tissue [26]. Therefore, we focus on the roles of FGF11 in goat BAT thermogenesis. As a member of intracellular FGFs, FGF11 has no obvious phenotype in FGF11 knockout mice [25]. FGF11 is a potential mediator of adipogenesis identified by transcriptome analysis of human adipose-derived stem cells during adipogenesis [32]. Moreover, FGF11 was upregulated during the adipogenic differentiation of brown adipocytes. We further employed isoprenaline (ISO) to induce cold stimulation in vitro, which resulted in increased expression of FGF11 in brown adipocytes. However, the functions and regulatory mechanisms of FGF11 in brown adipose thermogenesis are still unknown. In this study, FGF11 was enriched in BAT and plays regulatory roles in BAT thermogenesis. In addition, FGF11 induced UCP1 gene expression through regulating EBF2 transcription activity.

## 2. Results

### 2.1. Characterization and RNA-Seq Analysis of the Goat Perirenal Fat at 1 Day and 30 Days after Birth

**Figure 1:**
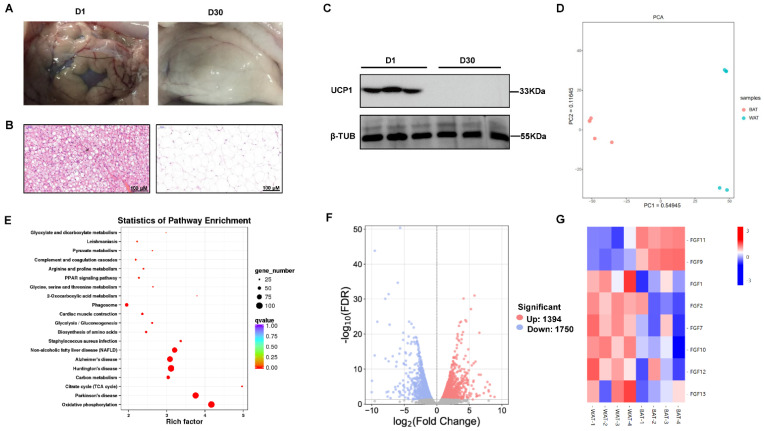
Characterization and identification of differential expression genes of the goat perirenal fat at 1 day (D1) and 30 days (D30) after birth. (**A**) The images show the transition in perirenal adipose tissue from D1 to D30. (**B**) Perirenal adipose tissue sections stained with hematoxylin and eosin (H&E). Scale bars, 100 μm. (**C**) Western blotting of UCP1 in BAT and WAT. (**D**) Principal component analysis (PCA) shows the relationship between BAT and WAT. The *x*-axis represents the 1st principal component, where 51.5% represents the contribution of the PC1 component to the sample. The *y*-axis represents the 2nd principal component, where 17.6% represents the contribution of the PC2 component to the sample. (**E**) Volcano plot showing differential gene expression between BAT and WAT. (**F**) Top 20 enriched KEGG pathways for differential genes of BAT and WAT using the KEGG pathway database. (**G**) The heatmap of FGF family genes differentially expressed in BAT and WAT.

To confirm the histological differences at two stages of perirenal fat, histological analysis was performed. The transition in the histological appearance of perirenal adipose tissue was evident from 1 day to 30 days (Figure 1A). The perirenal fat in 1 day goats showed characteristics of multilocular lipid droplets, whereas the perirenal fat in 30 day goats showed characteristics of unilocular lipid droplets in the sections with hematoxylin and eosin (H&E) (Figure 1B). Meanwhile, we found that UCP1 protein was specifically expressed in the perirenal fat of 1 day goats but was not expressed in the perirenal fat of 30 day goats (Figure 1C). These results indicated that the perirenal adipose tissue was BAT in newborn goats and transformed to WAT at 30 days after birth.

To understand the differences in transcript levels between BAT and WAT, we performed RNA-seq analysis. The clean data of all eight samples reached 16.47 Gb with percentages of Q30 bases at 93.76% and above. Principal component analysis (PCA) analyses showed a significant separation of gene expression between BAT and WAT using the first two principal component scores of PC1 and PC2 (54.9% and 11.6% of explained variance, respectively) (Figure 1D). Based on FDR < 0.05, and fold change (FC) > 2, a total of 3144 genes, including 1394 upregulated and 1750 downregulated genes, were determined in BAT compared with WAT (Figure 1E). The KEGG analysis showed that the differential expressed genes were enriched in top 20 pathways, which included oxidative phosphorylation, citrate cycle (TCA cycle), biosynthesis of amino acids, and PPAR signaling pathway (Figure 1F).

### 2.2. The Expression Pattern of FGF Family Genes between BAT and WAT

Previous studies have suggested important roles for the FGF family genes in adipogenesis and thermogenesis. Then, we performed further analysis of the differentially expressed FGF family genes. Interestingly, we found that *FGF11* and *FGF9* were significantly enriched in BAT compared with WAT (Figure 1G). Furthermore, the expression of *FGF1*, *FGF2*, *FGF7*, *FGF10*, *FGF12*, and *FGF13* genes were significantly lower in BAT compared with WAT. Other FGF family members, such as *FGF3*, *FGF6*, *FGF8*, *FGF14*, *FGF16*, *FGF17*, *FGF20*, *FGF21*, *FGF22*, and *FGF23* genes were also barely expressed with a count value lower than 50 and have no significantly different expression between BAT and WAT (Table S1).

### 2.3. Characterization and Expression Pattern of Goat FGF11 Gene

**Figure 2:**
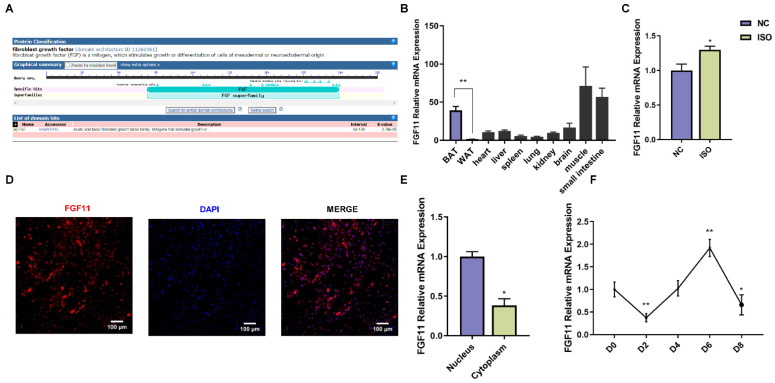
Identification and expression pattern of FGF11. (**A**) Predicted FGF11 protein domain diagram. (**B**) The expression of FGF11 in goat various tissues was determined by qPCR. (**C**) qPCR analysis of the FGF11expression under ISO simulated cold stimulation. (**D**) Immunocytochemistry of FGF11 (red), DAPI (blue). Scale bars, 100 μm. (**E**) FGF11 expression in the nucleus and the cytoplasm, respectively. (**F**) Expression levels of FGF11 during differentiation of brown adipocytes. Error bars represent standard error of mean (SEM), n = 6, * *p* < 0.05, ** *p* < 0.01.

The goat *FGF11* cDNA contains 678 bp of an open reading frame (ORF) encoding 225 amino acids with a protein molecular weight of 25.08 kDa. Protein domain prediction of FGF11 by conserved domain database (CDD) in NCBI showed that the protein encoded by *FGF11* belonged to the FGF family (Figure 2A). *FGF11* was widely expressed in tissues and the highest expression level of FGF11 was detected in BAT, muscle, and small intestine. In addition, the expression of *FGF11* in BAT was significantly higher than that in WAT (Figure 2B). To determine whether cold exposure induced the *FGF11* expression, we used isoprenaline (ISO) to simulate cold stimulation in vitro. ISO treatment significantly increased the expression of *FGF11* in brown adipocytes compared with control (Figure 2C). FGF11 immunocytochemistry indicated that FGF11 was primarily localized in the nuclear compartment (Figure 2D). Furthermore, the nuclear and cytoplasmic RNA extraction results showed that FGF11 expression was mainly located in the nucleus of brown adipocytes (Figure 2E). In addition, the expression of *FGF11* is highly expressed in preadipocytes, downregulated at day 2 of differentiation, and reached the highest level at day 6 of differentiation (Figure 2F).

### 2.4. Isolation and Identification of Goat Brown Adipocytes

**Figure 3:**
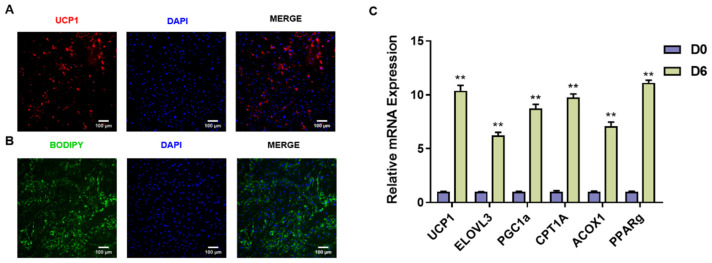
Isolation and identification of goat brown adipocytes. (**A**) Immunocytochemistry of UCP1 (red), DAPI (blue). Scale bars, 100 μm. (**B**) Amounts of lipid droplet in brown adipocytes by the BODIPY. Scale bars, 100 μm. (**C**) qPCR analysis of BAT marker genes expression in brown adipocytes after adipogenic differentiation at 6 days. Error bars represent standard error of mean (SEM), n = 6, ** *p* < 0.01.

To further investigate the impact of FGF11 on brown adipocytes, we isolated preadipocytes from brown adipose tissues of newborn goats. Then, brown adipocyte differentiation was induced using a standard differentiation cocktail. UCP1, a BAT marker, was used to evaluate the goat brown adipocytes. Subsequently, we conducted BODIPY staining and UCP1 immunocytochemistry on brown adipocytes (Figure 3A,B). In addition, the expression levels of BAT marker genes (*UCP1*, *ELOVL3*, *PGC1α*, *CPT1A*, *ACOX1*, and *PPARγ*) were significantly (*p* < 0.01) induced after adipogenic differentiation at 6 days (Figure 3C). These results suggested the successful induction of adipogenic differentiation and thermogenesis in goat brown adipocytes.

### 2.5. FGF11 Knockdown Inhibits Brown Adipocyte Differentiation and Thermogenesis

**Figure 4:**
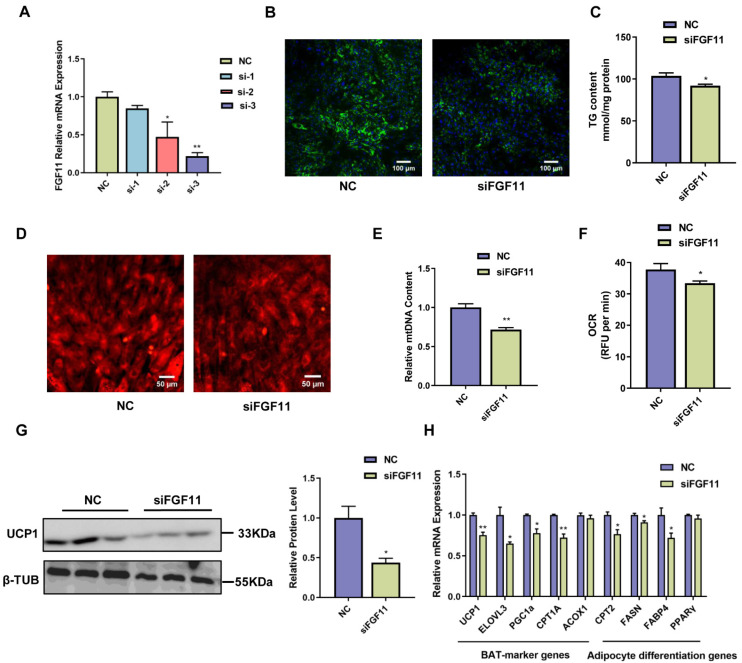
FGF11 knockdown inhibits brown adipocyte differentiation and thermogenesis. (**A**) Interference efficiency after FGF11 knockdown in brown adipocytes. (**B**) Amounts of lipid droplet in brown adipocytes after transfection with siFGF11 by BODIPY staining. BODIPY (green), DAPI (blue). Scale bars, 100 μm. (**C**) TG content in brown adipocytes after transfection with siFGF11. (**D**) Red fluorescence indicates mitochondrial content in brown adipocytes transfected with siFGF11. Scale bars, 50 μm. (**E**) Relative mitochondrial DNA content after transfection with siFGF11 in brown adipocytes. (**F**) Oxygen consumption assay for mature brown adipocytes after transfection with siFGF11. (**G**) Western blotting of UCP1 in brown adipocytes transfected with siFGF11. (**H**) qPCR analysis of related genes expression transfection with siFGF11 in brown adipocytes. Error bars represent standard error of mean (SEM), n = 6, * *p* < 0.05, ** *p* < 0.01.

To determine whether FGF11 affects brown adipocyte differentiation and thermogenesis, three siRNAs against FGF11 gene (siFGF11) were transfected into brown adipocytes and FGF11 was significantly decreased in brown adipocytes by transfection with si-3 (Figure 4A). Transfection of siFGF11 showed lower amounts of lipid droplets stained by the BODIPY staining compared to control (*p* < 0.05) (Figure 4B). The TG content was significantly (*p* < 0.05) decreased after transfection of siFGF11 (Figure 4C). FGF11 knockdown significantly reduced the number of mitochondria compared with control by staining with Mitotrcker Red and mitochondrial DNA content assay (*p* < 0.01) (Figure 4D,E). The mitochondrial oxygen consumption ratio was also significantly decreased after FGF11 knockdown (Figure 4F). In addition, the expression of UCP1 protein was significantly decreased after FGF11 knockdown compared with control (Figure 4G). Moreover, the expression levels of adipocyte differentiation genes (*FASN*, *FABP4*, and *PPARγ*) were significantly (*p* < 0.05) inhibited by FGF11 knockdown. In addition, the BAT marker genes, including *UCP1*, *ELOVL3*, *PGC1α*, *CPT1A*, and *CPT2* were significantly (*p* < 0.05) downregulated in brown adipocytes compared to control (Figure 4F). Taken together, these results indicated that *FGF11* plays a critical role in differentiation and thermogenesis of brown adipocytes.

### 2.6. FGF11 Overexpression Promotes Brown Adipocyte Differentiation and Thermogenesis

**Figure 5:**
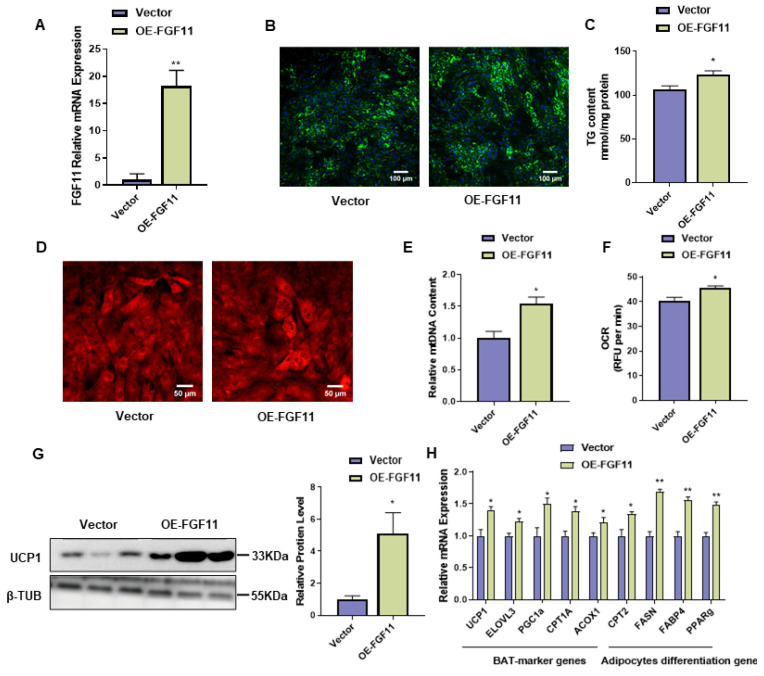
FGF11 overexpression promotes brown adipocytes thermogenesis. (**A**) Interference efficiency after FGF11 overexpression in brown adipocytes. (**B**) Amount of lipid droplets in brown adipocytes after transfection with FGF11-pEGFP-N1, assessed by BODIPY staining. BODIPY (green), DAPI (blue). Scale bars, 100 μm. (**C**) TG content in brown adipocytes after transfection with siFGF11. (**D**) Red fluorescence indicates mitochondrial content in brown adipocytes transfected with FGF11-pEGFP-N1. Scale bars, 50 μm. (**E**) Relative mitochondrial DNA content after transfection with siFGF11 in brown adipocytes. (**F**) Oxygen consumption assay to mature brown adipocytes after transfected with FGF11-pEGFP-N1. (**G**) Western blotting of UCP1 in brown adipocytes transfected with FGF11-pEGFP-N1. (**H**) qPCR analysis of related gene expression after transfection with FGF11-pEGFP-N1 in brown adipocytes. Error bars represent standard error of mean (SEM), n = 6, * *p* < 0.05, ** *p* < 0.01.

To investigate whether FGF11 overexpression is involved in brown adipocyte differentiation, brown adipocytes were transfected with the FGF11-pEGFP-N1 overexpression vector. The expression level of *FGF11* was highly upregulated by *FGF11* overexpression (Figure 5A). Compared to the control group, the overexpression of *FGF11* increased (*p* < 0.05) the lipid accumulation of brown adipocytes, as detected by BODIPY staining (Figure 5B). The TG content was significantly increased after FGF11 overexpression (Figure 5C). As shown in Figure 5D,E, the number of mitochondria was significantly (*p* < 0.05) increased and the mitochondrial oxygen consumption ratio was also significantly increased (*p* < 0.05) (Figure 5F). The UCP1 protein was significantly (*p* < 0.05) increased after FGF11 overexpression (Figure 5G). In addition, the expression of adipocyte differentiation genes (*FASN*, *FABP4*, and *PPARγ*) was significantly upregulated after FGF11 overexpression (*p* < 0.05). Furthermore, the BAT marker genes (*UCP1*, *ELOVL3*, *PGC1α*, *CPT1A*, *ACOX1,* and *CPT2*) were upregulated (*p* < 0.05) compared with control cells after FGF11 overexpression (Figure 5H).

### 2.7. FGF11 Has No Effect on White Adipocyte Differentiation and Thermogenesis

**Figure 6:**
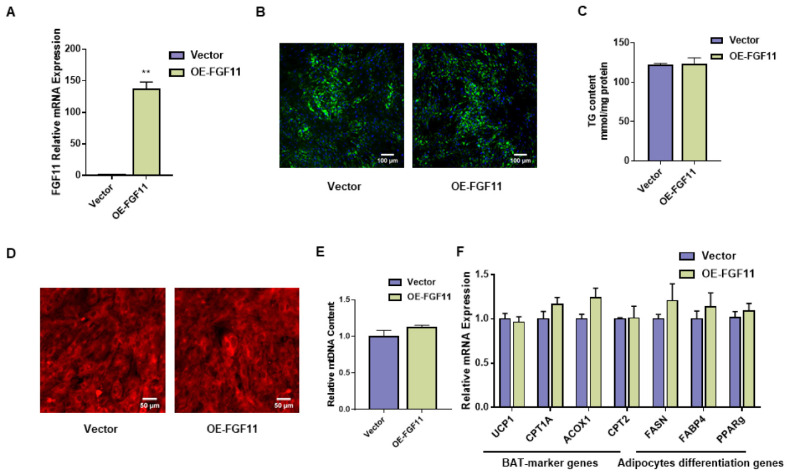
FGF11 overexpression did not affect white adipocyte thermogenesis. (**A**) Overexpression efficiency after transfection with FGF11-pEGFP-N1 in white adipocytes. (**B**) Amounts of lipid droplet in white adipocytes after FGF11 overexpression, assessed by BODIPY staining. BODIPY (green), DAPI (blue). Scale bars, 100 μm. (**C**) TG content in brown adipocytes after transfection with FGF11-pEGFP-N1. (**D**) Red fluorescence of white adipocytes, assessed by Mito-Tracker Red. Scale bars, 50 μm. (**E**) Relative mitochondrial DNA content after FGF11 overexpression in brown adipocytes. (**F**) qPCR analysis of related genes expression in white adipocytes. Error bars represent standard error of mean (SEM), n = 6, ** *p* < 0.01.

To further explore the roles of FGF11 in white adipocytes, siFGF11 and FGF11-pEGFP-N1 overexpression vectors were transfected into white adipocytes, respectively. As shown in Figure 6A, FGF11 expression was distinctly upregulated by transfection of FGF11-pEGFP-N1. However, there is not a detectable difference in lipid accumulation (Figure 6B). In addition, the number of mitochondria was not changed. The qPCR result showed that there were no significant changes in BAT thermogenesis and adipocyte differentiation genes (Figure 6C,D). Furthermore, transfection of siFGF11 revealed that there were no distinct changes in lipid accumulation, mitochondrial content, and the expression of related genes after FGF11 knockdown (Figure S1).

### 2.8. FGF11 Induced UCP1 Gene Expression through Regulating EBF2 Binding to UCP1 Promoter

**Figure 7:**
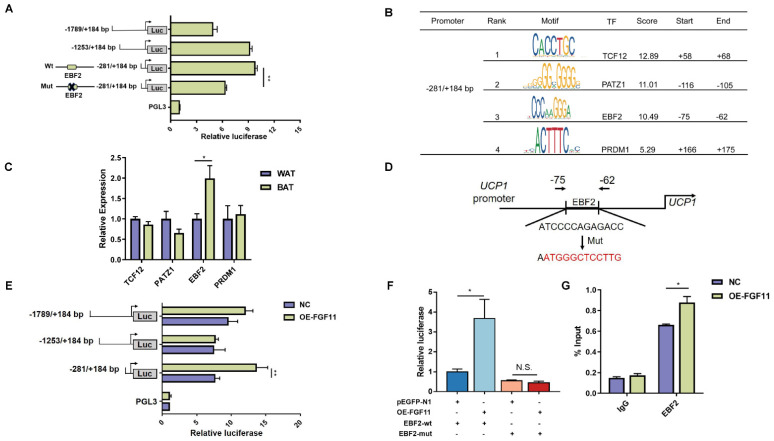
FGF11 regulated UCP1 expression by EBF2. (**A**) Four UCP1 promoter fragments were transfected into brown adipocytes for 48 h. (**B**) Binding site prediction of EBF2 on the UCP1 proximal promoter was performed by Jasper database. (**C**) The expression levels of TCF12, PATZ1, EBF2, and PRDM1 between WAT and BAT. (**D**) Twelve site-specific mutations of EBF2 biding site to construct the mutant promoter. (**E**) Transfection with EBF2-Mut vector into brown adipocytes for 48 h. (**F**) Four truncated UCP1 promoter fragments were transfected into brown adipocytes together with FGF11-pEGFP-N1 for 48 h. (**G**) Transfection with Luc-EBF2-WT and Luc-EBF2-Mut in brown adipocytes. G Enrichment of EBF2 at UCP1 promoter was detected by ChIP-qPCR. Error bars represent standard error of mean (SEM), n = 6, * *p* < 0.05, ** *p* < 0.01.

To further verify how FGF11 regulates the *UCP1* expression, four deletion segments of the *UCP1* gene promoter constructed to luciferase reporter PGL3-basic vector were transfected into brown adipocytes. We found that all four deletion segments showed high activity compared with pGL3-basic (Figure 7A). The −281 to +184 bp sequence was analyzed with Jasper database (https://jaspar.genereg.net/ (access on 15 January 2023)) to identify possible transcription factor binding sites on the *UCP1* gene promoter. We found that the TCF12, PATZ1, EBF2, and PRDM1 binding sites (Figure 7B). We further verified the expression levels of genes between BAT and WAT. From these transcription factors, the expression of EBF2 was significantly upregulated (*p* < 0.05) in BAT. However, TCF12, PATZ1, and PRDM1 expression did not change between BAT and WAT (Figure 7C). In addition, EBF2 was previously characterized to promote the expression of thermogenic genes in adipose tissue [33]. Therefore, it is important to explore whether EBF2 regulates the UCP1 expression in brown adipocytes.

To investigate whether EBF2 actually binds to the *UCP1* promoter region to affect *UCP1* expression, we made twelve site-specific mutations at this binding site and constructed them into the pGL3 vector to examine the significance of the EBF2 binding site (Figure 7D). The luciferase activity of −281 to +184 bp sequence was significantly decreased after the EBF2 binding site mutation (Figure 7A). Next, the four deletion segments of the *UCP1* promoter vector were co-transfected with FGF11-pEGFP-N1 into brown adipocytes. As shown in Figure 7E, the −281 to +184 bp sequence and −635 to +184 bp sequence showed obvious promotion by co-transfecting with FGF11-pEGFP-N1. The activity of the −281 to +184 bp sequence was significantly (*p* < 0.05) downregulated by transfecting with the mutation vector and the activity of the mutation segment did not increase by co-transfection with FGF11-pEGFP-N1 (Figure 7F). To investigate whether FGF11 regulated EBF2 binding to *UCP1* gene promoter, the ChIP-qPCR assay was used to determine the enrichment of EBF2 in the *UCP1* gene promoter. We found that FGF11 increased EBF2 binding to *UCP1* gene promoter (Figure 7G). Together, these results suggest that EBF2 binds and activates the *UCP1* gene promoter regulated by FGF11.

## 3. Discussion

Lambs are generally born in a low-temperature environment, and the non-shivering thermogenesis of BAT is important for maintaining their central body temperature [21]. It has been reported that BAT produces half the amount of heat to maintain a central temperature in newborn lambs [34]. BAT thermogenesis is regulated through norepinephrine released from the sympathetic nervous system that activates thermogenesis during cold stress in brown adipocytes [35]. Among the FGF family, several members have been proven to play an important role in adipose tissue thermogenesis and differentiation. FGF6 and FGF9 can promote UCP1 expression in brown fat upon cold stimulation. FGF1 promotes human preadipocytes differentiation and proliferation. Upon acute cold exposure, FGF21 increases UCP1 expression and BAT thermogenesis to keep core body temperature in mice [36]. Our transcriptome data revealed that among the FGF family members, only the expressions of FGF9 and FGF11 were upregulated in BAT. However, the mechanism underlying the effect of FGF11 on BAT thermogenic function has not been investigated. Our study reveals that FGF11 plays an important role in promoting goat brown adipocytes and the expression genes related to thermogenesis.

A previous study has shown that the expression of FGF11 increases during the differentiation of human adipose-derived stem cells [32]. In this study, our finding showed that FGF11 expression is higher in BAT than in WAT. FGF11 expression is upregulated during brown adipocyte differentiation and isoprenaline treatment increased the expression of *FGF11* in brown adipocytes. These results indicates that FGF11 may play important roles in the brown adipocyte differentiation and thermogenesis. In addition, FGF11 was demonstrated to induce the differentiation and adipogenesis of 3T3-L1 preadipocytes by regulating the expression of PPARγ [37]. Our finding shows that FGF11 promotes the expression of adipogenesis genes in brown adipocytes. However, there are no significant changes in the expression of adipogenesis genes of white adipocytes by overexpression of *FGF11*. This may be because of differences in the regulation of adipocytes differentiation between goat and mouse white adipocytes. In addition, we noticed that the FGF11 protein is not detected in the adipose tissue according to the human protein atlas (https://www.proteinatlas.org/ (accessed on 15 June 2023)), whereas the highest expression level of FGF11 was detected in goat brown adipose tissue. We further found that FGF11 promoted goat brown adipocyte development and thermogenesis. This provides evidence for differences in the regulation roles of FGF11 among species.

The subcellular location indicated that FGF11 was mainly expressed in the nucleus. These results proved that FGF11 is a member of intracellular FGFs and may play important roles in the genes transcriptional regulation. Meanwhile, FGF11 regulated the expression of UCP1, which is located in the inner mitochondrial membrane of brown adipocytes, which can dissipate the energy stored in the mitochondrial electrochemical gradient as heat upon ATP synthesis [38]. A previous study has shown that inhibition of FGF11 in the hypothalamus prevented diet-induced obesity and enhanced BAT thermogenesis via the sympathetic nervous system [39]. In the present study, FGF11 promoted the expression of the UCP1 protein and an EBF2 element was responsible for UCP1 promoter activity. Additionally, FGF11 induced *UCP1* gene expression through regulating EBF2 transcription activity. Early B-cell factor 2 (EBF2) is a key transcriptional regulator and it is selectively expressed in brown and beige adipose tissue; it recruits PPARγ to brown adipose-selective gene targets to induce brown adipocyte differentiation [40]. EBF2 controls the process of browning in WAT, and EBF2 knockout in mice affected beige adipocyte development and function [41]. UCP1 expression is found to be almost abolished in mice with an adipocyte-specific EBF2 mutation [42]. Our results demonstrate that the EBF2 element was responsible for UCP1 promoter activity in goat brown adipocytes. In addition, FGF11 regulated UCP1 expression by regulating EBF2 transcription activity.

## 4. Materials and Methods

### 4.1. Animals

All research involving animals was conducted according to the approved protocols of the Institutional Animal Care and Use Committee at the College of Animal Science and Technology, Sichuan Agricultural University, Sichuan, China. The female Chuanzhong black goats were raised at the breeding center of the Sichuan Agricultural University, Ya’an, China. In this study, the perirenal adipose tissues were collected from female goat kids at 1 day and 30 days afterbirth (4 individuals at each stage). Goat kids were allowed ad libitum to water and feed breast milk with hay supplementation twice daily. All goats fasted overnight before being injected with su-mian-xin (0.1 mL/kg BW) (Shengda, Changchun, China), which is an animal anesthetic. After slaughtering, perirenal adipose tissues were immediately sampled, and frozen at −80 °C.

### 4.2. Histology Analysis

Perirenal adipose tissues at 1 day and 30 days afterbirth were fixed in 4% paraformaldehyde and then embedded in paraffin. For hematoxylin and eosin (H&E) staining, the sections were stained with hematoxylin (Solarbio, Beijing, China). These sections were observed by a microscope (Olympus Optical, Tokyo, Japan).

### 4.3. RNA-Seq Analysis

Total RNA was extracted by TRIzol (Invitrogen, Carlsbad, CA, USA). Next, we used NEBNext^R^ Ultra^TM^ RNA Library Prep Kit (NEB, MA, USA) to generate the eight sequencing libraries, which then were sequenced by the Hiseq X Ten (Illumina, San Diego, CA, USA). The raw reads that contained an adapter and low-quality reads were removed to obtain clean reads through trim_galore. Trim_galore is a wrapper tool around Cutadapt and FastQC, which is suitable for all high-throughput sequencing and can remove low-quality bases and streets to obtain clean reads. The clean reads were aligned to the goat reference genome Capra_hircus ARS1 by HISAT2 [43]. The reads mapped to the goat reference genome were assembled and quantified by String Tie [44]. Differential expression analysis between two stages was analyzed using the edgeR. The significance of differentially expressed genes were set at a false discovery rate (FDR) < 0.05 and fold change > 2 [45]. The differentially expressed genes were used for KEGG pathway analysis [46]. RNA-seq data are available at the Sequence Read Archive (SRA) database, accession number PRJNA547456.

### 4.4. Brown and White Preadipocytes Culture

The brown preadipocytes were isolated from goat perirenal BAT at 1 day after birth. After washing with phosphate-buffered saline (PBS), the BAT was digested with type Ⅱ-collagen enzyme (2 mg/mL) (BioFrox, Guangzhou, China) for 25 min at 37 °C, followed by filtration through a 70 µm filter. After centrifugation at 1500× *g* for 5 min, we added the medium containing 10% FBS (Gibco, Emeryville, CA, USA), streptomycin, penicillin, and DMEM/F12 (Meilunbio, Dalian, China), and transferred it to the culture bottle under conditions of 5% CO_2_ and 37 °C.

Primary white adipocytes were obtained from the perirenal WAT at 30 days after birth. Brown and white adipocytes were cultured in media supplemented with 10% FBS, 860 nM insulin (Solarbio, Beijing, China), 1 nM triiodothyronine(T3) (Selleck, Houston, TX, USA), 1 μM rosiglitazone (Selleck, TX, USA), 1 µM dexamethasone (MCE, Shanghai, China), and 0.5 mM IBMX (Sigma, Marlborough, MA, USA) for 2 days. Cells were then treated in maintenance media (2% FBS, 860 nM insulin, and 1 nM T3) for 6 days.

### 4.5. Vector Construction and Cell Transfection

The coding sequences of *FGF11* were inserted into pEGFP-N1 (Clontech, Palo Alto, CA, USA) using T4 ligase (Takara, Tokyo, Japan) with Sam Ⅰ (Takara, Tokyo, Japan) and Hind Ⅲ (Takara, Tokyo, Japan). The siRNAs against the *FGF11* gene (si-1: GCATTTCACAGCTGAGTGT, si-2: GCGTCTTTGAGAATTACTA, si-3: GTACGCCTCTGCTCTCTAT) and the negative control siRNAs were synthesized from RIOBIO (Guangzhou, China). White and brown adipocytes were transfected with siRNAs using Lipofectamine 3000 after 2 days of differentiation. For *FGF11* overexpression, white and brown adipocytes were transfected with FGF11-pEGFP-N1 using lipofectamine 3000 (Invitrogen, CA, USA) after 2 days of differentiation. To determine the subcellular localization of FGF11, DAPI (Sangon, Shanghai, China) was added to the plate to locate the nucleus and then observed by microscopy (Nikon, Tokyo, Japan).

### 4.6. BODIPY and Mitochondrial Staining

Adipocytes were stained with BODIPY-493/503 (Invitrogen, CA, USA). The adipocytes were fixed in 4% paraformaldehyde for 30 min and then washed three times with PBS. BODIPY (5 μM) was stained in the dark at room temperature for 20 min. Next, the BODIPY staining solution was aspirated, and DAPI staining was applied for 5 min. The cells were then observed under a fluorescence inverted microscope (Nikon, Tokyo, Japan). For mitochondrial staining, 1 µL Mito-Tracker Red CMXRos storage solution (Beyotime, Shanghai, China) was incubated in brown and white adipocytes at 37 °C for 30 min, and finally observed by microscopy (Nikon, Tokyo, Japan).

### 4.7. Quantitative Analysis of Triglycerides

We employed a triglyceride detection kit (Nanjing Jiancheng, Nanjing, China) to quantify the triglyceride content in adipocytes. The adipocytes were collected in a 6-well plate and then treated with the reagents from the kit. The absorbance was measured using a Varioskan LUX Microplate Reader (Thermo Fisher Scientific, Waltham, MA, USA) at 510 nm and was subsequently normalized to the protein content.

### 4.8. Immunocytochemistry

We conducted immunocytochemistry analysis on brown and white adipocytes. The adipocytes were initially fixed with 4% paraformaldehyde for 15 min and subsequently incubated with 0.25% Triton X-100 (Solarbio, Beijing, China) at room temperature for 10 min. Subsequently, the washed cells were incubated overnight at 4 °C with the following primary antibodies: rabbit anti-UCP1 (dilution 1:100, Cell Signaling Technologies, Danvers, MA, USA) and rabbit anti-FGF11 (dilution 1:250, Bioss, Beijing, China). The cells were incubated with the secondary antibody (Cy3 Goat Anti-Rabbit IgG) diluted at 1:500 and maintained at 37 °C for 1 h. Finally, the cells were stained with DAPI and observed under a microscope.

### 4.9. Oxygen Consumption Assays

After 2 days of differentiation, adipocytes in a 96-well plate were transfected with overexpression vectors or siRNAs. After 6 days of transfection, the oxygen consumption rate (OCR) was measured using the Extracellular Oxygen Consumption Assay Kit (Abcam, Cambridge, UK), guided by the manufacturer indications.

### 4.10. RNA Extraction and qPCR

Total RNA was extracted from adipocytes and tissues by TRIzol (Invitrogen, CA, USA) and 1 µg RNA was amplified to cDNA with the Reverse Transcription kit (ABCLONAL, Wuhan, China). The qPCR was carried out with 5 μL of Genious 2X SYBR Green Fast qPCR Mix (ABCLONAL, Wuhan, China), 0.8 μL of cDNA, 0.4 μL of primers (Table S2), and 3.4 μL ddH_2_O by the Bio-Rad CFX96 instrument (Bio-Rad, Hercules, CA, USA). Relative mRNA expression was determined by the method of ΔCt. Our previous study showed that PFDN5 is one of the most stable internal genes between BAT and WAT [47]. In this study, PFDN5 was used as an internal reference gene. To determine the subcellular localization of FGF11, nuclear and cytoplasmic RNA was extracted using the cytoplasmic and nuclear RNA purification kits (Norgen Biotek, Thorold, ON, Canada). 

### 4.11. Mitochondrial DNA Content Assay

Total DNA was extracted by TIANamp Genomic DNA Kit (Tiangen, Beijing, China). The relative level of mtDNA content was detected by qPCR analysis. Differences in CT values between ND1 and nuclear *TBP* were used to calculate the relative level of mtDNA content. qPCR was amplified as described above using primers in Table S1.

### 4.12. Luciferase Assays

The *UCP1* gene promoter sequences were truncated into four segments and constructed into pGL3-Basic. Meanwhile, the sequence of EBF2 mutation sites were synthesized and constructed into pGL3-Basic. FGF11-pEGFP-N1 was co-transfected with the vector of UCP1 promoter and the vector of EBF2 mutation sites by Lipofectamine 3000 (Invitrogen, CA, USA) when the brown preadipocytes reached 80% confluency. After 2 days, luciferase activity was detected by the TransDetect^®^ Double-Luciferase Reporter Assay Kit (TransGen Biotech, Beijing, China).

### 4.13. Western Blotting

The proteins used in the experiments were extracted using the protein extraction kit (Bestbio, Shanghai, China). The following primary antibodies were used: dilution 1:1000 for rabbit anti-UCP1 (Cell Signaling Technologies, MA, USA), and dilution 1:3000 for rabbit anti-β-Tubulin (Abclonal, Wuhan, China). The primary antibodies were incubated at 4 °C overnight. HRP-labeled goat anti-rabbit secondary antibody was diluted at 1:1000 for 1.5 h at 37 °C. Finally, the proteins were detected by the ECL detection system (Beyotime, Shanghai, China).

### 4.14. ChIP-qPCR

The coding sequences of *EBF2* were inserted into pEGFP-N1, and the coding sequences of *FGF11* were inserted into PCDNA3.1 (Clontech, Palo Alto, CA). EBF2-pEGFP-N1 was co-transfected with FGF11-PCDNA3.1 into brown adipocytes by Lipofectamine 3000 after 2 days of differentiation. The ChIP-qPCR assay was detected 48 h after transfection by the ChIP Assay Kit (Beyotime, Shanghai, China). Firstly, the cells were cross-linked using 1% formaldehyde solution for 10 min at room temperature, and the reaction was then stopped by glycine solution. Then, the cells were lysed on ice by adding SDS lysis buffer containing 1 M PSMF, and the nuclei were lysed. The chromatin was sheared into an average size of 400–800 bp by sonication. A part of the supernatant was used as input control, and the rest was split into two aliquots, and anti-IgG antibody (ABCLONAL, Wuhan, China) and anti-GFP antibody (Proteintech, Chicago, IL, USA) were added respectively for 12 h at 4 °C. The chromatin–antibody complex was pelleted by Protein A+G Agarose/Salmon Sperm DNA. Cross-links between chromatin and protein were reversed at 65 °C for 4 h. Precipitated DNA can be used for qPCR after purification. ChIP-qPCR were amplified as described above (Table S2 for primers).

### 4.15. Statistical Analysis

All statistical analyses were performed through Prism 7.0 (GraphPad, San Diego, CA, USA). All data were represented as mean ± SEM. Two groups of sample data were analyzed by the unpaired Student’s *t* test, and three groups of sample data were analyzed using one-way ANOVA.

## 5. Conclusions

We performed transcriptome analysis between goat BAT and WAT and found that *FGF11* was enriched in BAT. Meanwhile, FGF11 promoted differentiation and thermogenesis in brown adipocytes but not in white adipocytes. Furthermore, the FGF11–EBF2–UCP1 axis was essential for the brown adipocyte thermogenic gene program. These results provide a reference for the study of FGF11 and transcription factor regulatory networks in brown adipocyte development and thermogenesis.

## Data Availability

RNA-seq data are available at the Sequence Read Archive (SRA) database, accession number PRJNA547456.

## Supplementary Materials

The following supporting information can be downloaded at: https://www.mdpi.com/article/10.3390/ijms241310838/s1.
