# Supplementary material for: Transcriptomic Analysis Reveals Fibroblast Growth Factor 11 (FGF11) Role in Brown Adipocytes in Thermogenic Regulation of Goats
Source: Int J Mol Sci. 2023 Jun 29;24(13):10838. doi: 10.3390/ijms241310838 (PMC10341626; doi:10.3390/ijms241310838)
Supplement: Supplementary file 1 [file ijms-24-10838-s001.zip › Figure S1.pdf]

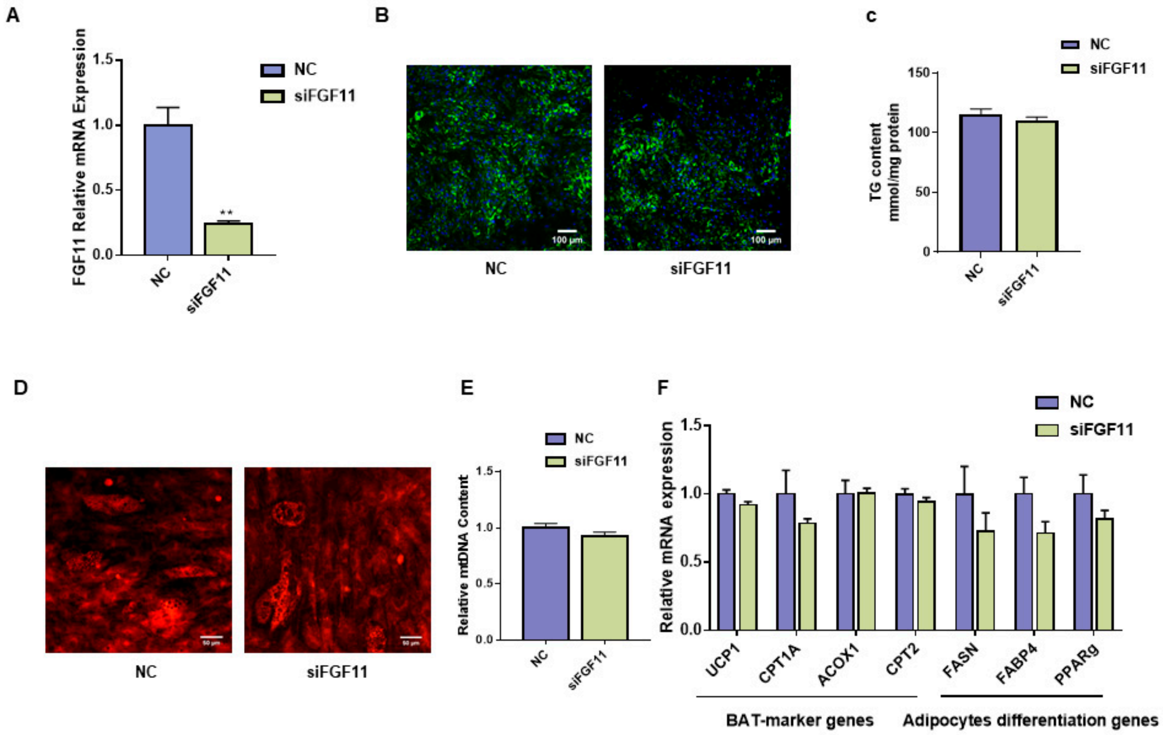

Figure S1 FGF11 knockdown did not affect white adipocytes thermogenesis. A Interference efficiency after FGF11 knockdown in white adipocytes. **B** Amounts of lipid droplet in white adipocytes after transfection with siFGF11 by BODIPY staining. BODIPY (green), DAPI (blue). Scale bars, 100 μm. **C** TG content in brown adipocytes after transfection with siFGF11. **D** Red fluorescence of white adipocytes by Mito-Tracker Red. Scale bars, 50 μm. **E** Relative mitochondrial DNA content after transfection with siFGF11 in brown adipocytes. **F** qPCR analysis of related genes expression transfection with siFGF11 in white adipocytes. Error bars represent standard error of mean (SEM), n = 6, *$p < 0.05$, **$p < 0.01$.
