# Supplementary material for: Transcriptomic Analysis Reveals Fibroblast Growth Factor 11 (FGF11) Role in Brown Adipocytes in Thermogenic Regulation of Goats
Source: Int J Mol Sci. 2023 Jun 29;24(13):10838. doi: 10.3390/ijms241310838 (PMC10341626; doi:10.3390/ijms241310838)
Supplement: Supplementary file 1 [file ijms-24-10838-s001.zip › Table S2.pdf]

Table S2. Primers used for qPCR.

Gene symbol	Sequence 5′-3′	Tm(°C)	Size(bp)
UCP1	ATCTCAGCGGGCCTAACAAC<br>CTTTCCAAAGCCCCGTCAAG	61.4	178
PGC1α	CCACAAATGATGACCCTC<br>GGTTTGGCTTGTAGATGTT	59.0	103
ELOVL3	ATGAGGCCCTTTTTGGAGGAG<br>CACATCCTCAGTGTCCCGAA	61.4	182
PPARγ	GTGTCACTCCTGAACGAAAT<br>GGAAATGCTGGAGAAGTCAA	61.4	156
FASN	CTGCTCAGTGGGCTCCTCA<br>TGGCGGTCAGTGGCTATGT	61.4	187
FABP4	ACTGGGATGGGAAATCAACC<br>CCTTGGCTTATGCTCTCTCG	59.0	117
CPT1A	CGTCGCCTTCCAGTTCACAG<br>CACAACCACGATGAGCCAACT	61.4	181
ACOX1	CGTAGCCAGCGTTATGAGGT<br>TCCAGGCAGGCATGAAGAAG	61.4	220
CPT2	GTGGACTCGGCTGTGTTCT<br>TGCCATCCTTGGCTATAAT	61.4	142
PFDN5	AAGACAGCTGAGGATGCCAAG<br>AGGGTTGTGAGCTGCTGAATC	61.4	155
UCP1 promoter 1	CTGAGCTCGCGCTTGTTT<br>GTCCCGAAGGTGGAAGTT	60	465
UCP1 promoter 2	TTCACCACACTGCCATAC<br>GTCCCGAAGGTGGAAGTT	60	819
UCP1 promoter 3	CCACATCAGATGGAACCA<br>GTCCCGAAGGTGGAAGTT	60	1437
UCP1 promoter 4	CAATACTTTGGCCACCTG<br>GTCCCGAAGGTGGAAGTT	60	1972
ChIP-qPCR	CATCGAGGGTAGAGAGTAGA<br>CCTCCGCGGATGACTTATAT	61.4	159
ND1	CCGAAAAGGTCCAAACGTCG<br>ATTGAGGATGTGGCAGGTCG	61.4	105
Nuclear TBP	CAGTGCAGATTCTGTCCACG<br>CAGTGCAGATTCTGTCCACG	61.4	118
